# What Do Placebo and Nocebo Effects Have to Do With Health Equity? The Hidden Toll of Nocebo Effects on Racial and Ethnic Minority Patients in Clinical Care

**DOI:** 10.3389/fpsyg.2021.788230

**Published:** 2021-12-23

**Authors:** Hailey E. Yetman, Nevada Cox, Shelley R. Adler, Kathryn T. Hall, Valerie E. Stone

**Affiliations:** ^1^Department of Medicine, Brigham and Women’s Hospital, Harvard Medical School, Boston, MA, United States; ^2^Penn State College of Medicine, Hershey, PA, United States; ^3^Osher Center for Integrative Health, University of California, San Francisco, San Francisco, CA, United States; ^4^Department of Medicine, Harvard Medical School, Boston, MA, United States

**Keywords:** placebo and nocebo effects, health inequities, communication, mistrust, perceived discrimination

## Abstract

A placebo effect is a positive clinical response to non-specific elements of treatment with a sham or inert replica of a drug, device, or surgical intervention. There is considerable evidence that placebo effects are driven by expectation of benefit from the intervention. Expectation is shaped by a patient’s past experience, observations of the experience of others, and written, verbal, or non-verbal information communicated during treatment. Not surprisingly, expectation in the clinical setting is strongly influenced by the attitude, affect, and communication style of the healthcare provider. While positive expectations can produce beneficial effects, negative information and experiences can lead to negative expectations, and consequently negative or nocebo effects. Key components identified and studied in the placebo and nocebo literature intersect with factors identified as barriers to quality care in the clinical setting for Black patients and other patients of color, including poor patient-clinician communication, medical mistrust, and perceived discrimination. Thus, in the context of discrimination and bias, the absence of placebo and presence of nocebo-generating influences in clinical settings could potentially reinforce racial and ethnic inequities in clinical outcomes and care. Healthcare inequities have consequences that ripple through the medical system, strengthening adverse short- and long-term outcomes. Here, we examine the potential for the presence of nocebo effects and absence of placebo effects to play a role in contributing to negative outcomes related to unequal treatment in the clinical encounter.

## Introduction

### Placebo and Nocebo Effects

A placebo effect is a positive outcome in response to an inert treatment or intervention. The mechanism underlying the placebo response has captivated and bewildered clinicians and researchers for decades. To understand why patients respond to seemingly inert treatments, placebo researchers investigated components of the clinician-patient encounter and found that positive *expectations*, both conscious and unconscious, are key drivers of placebo effects (Atlas, 2021). The word “placebo” is derived from the Latin *placere*, “to please.” While placebo effects are widely known to be salubrious, their opposite, nocebo effects, are not as well known. Nocebo effects are negative outcomes that are induced by negative expectations, both conscious and unconscious. The term, derived from the Latin word *nocere*, “to harm,” was initially coined in 1961 to describe the phenomenon of adverse events occurring in clinical trial participants randomized to placebo (Kennedy, 1961). Today the term is used in many contexts, one of which is to describe components of the clinical encounter that might negatively affect treatment outcomes.

The nocebo effect was first reported in blinded clinical trials with placebo controls. To the surprise of investigators, participants given placebo in these trials reported side effects, some of which were very closely related to the expected side effects of the active drug. As there were no obvious biological impacts of the placebo pill itself, investigators ascribed the adverse events to psychologically induced negative effects arising from learning about the potential side effects of the active treatment. This phenomenon continues to pose a challenge to drug developers in their attempts to develop new treatments for depression (Kirsch and Sapirstein, 1998; Khan et al., 2002) Alzheimer’s disease (Lim et al., 2020), and heart disease prevention (Wood et al., 2020).

The information conveyed by clinicians about a newly prescribed therapy can also shape patients’ experiences. Often, if the clinician emphasizes the side-effects of a treatment, the patient is more likely to experience those side-effects (Silvestri et al., 2003; Rief et al., 2006; Doering et al., 2014). Other influencing factors may include subtle cues derived from the posture and facial expressions of the clinician and whether they establish eye contact or physical touch. When these subtle cues are deemed positive, they can enhance placebo effects (Kaptchuk et al., 2008), but when they are negative, the opposite is true and they can induce nocebo effects (Jensen et al., 2012).

Expectations are considered to be key drivers of placebo and nocebo effects and can arise from the patient’s past experience, direct information conveyed to the patient by the clinician, and subtle cues in the patient-clinician interaction. If a patient experienced nausea with a treatment in the past, they might expect to again feel nauseous the next time they are prescribed that treatment. Patients with cancer, for example, may experience “anticipatory nausea” before chemotherapy treatments actually begin upon entering the facility where they usually have chemotherapy treatment, or even entering a room with walls painted the same color as the one in which they were usually treated (Andrykowski, 1988; Kamen et al., 2014; Colloca and Barsky, 2020). Many experts consider these negative symptoms to be a product of conditioning from prior experiences. The patient repeatedly feels nauseous during chemotherapy, and over time they are conditioned to associate the treatment room with nausea, even if the room itself is not causing the symptom. Patients that had negative experiences or experienced discrimination in the clinical encounter have different expectations, including increased mistrust and expectations of experiencing discrimination again in the future, than those that have had mostly positive experiences in the clinical encounter (Atlas, 2021; Hall et al., 2021). The implication is that patients could be conditioned to anticipate future poor outcomes with negative encounters.

Due to the ethical imperative of clinicians to “do no harm,” and the general aversion to deception of the patient, the study of intentionally induced nocebo effects is limited (Wolters et al., 2019). Intentional introduction of negative experiences can have lasting adverse effects on patients and is therefore highly regulated. Thus, a substantial portion of nocebo research occurs in laboratory settings. Despite the limitations in placebo and nocebo studies, it is clear that the absence of placebo and presence of nocebo-promoting factors can result in negative expectations that increase the experience of pain (Scott et al., 2008), reduce treatment efficacy (van Laarhoven et al., 2011), and compromise short- and long-term health outcomes.

The links between race/ethnicity, health inequities, and placebo and nocebo effects are not well studied (Friesen and Blease, 2018). Four components of the clinical encounter that are considered to mediate suboptimal outcomes by experts in health inequities include poor communication, medical mistrust, perceived discrimination, and racial discordance. Here, we examine these components through a placebo/nocebo lens to determine how the two literatures might converge to better explain how these factors lead to suboptimal outcomes. Further, we investigate the potential for nocebo and placebo mechanisms to play a role in exacerbation of negative outcomes due to unequal treatment in the clinical encounter and support this by connecting evidence from the existing placebo, nocebo, and health inequity literature.

### Unequal Treatment

Health inequities research has demonstrated that patients’ race and ethnicity can significantly impact the care that they receive and subsequent clinical outcomes. The report *Unequal Treatment*: *Confronting Racial and Ethnic Disparities in Health Care* published by the Institute of Medicine concluded that “race and ethnicity remain significant predictors of quality of health care received” (Nelson, 2002). In 2017, a report from the Centers for Disease Control (CDC) reported that Black Americans were more likely to die from diabetes, heart disease, and cancer than their White counterparts (Hamel et al., 2020). More recently, the Kaiser Family Foundation’s (KFF) 2020 Report confirmed that Black Americans have more negative experiences in the health care setting and more significant barriers to accessing healthcare than White Americans (Hamel et al., 2020). In a February 2021 report from the CDC, the life expectancy for non-Hispanic Black Americans was 72 compared to 78 years for non-Hispanic White Americans (Seible et al., 2021). In the United States, infant mortality for Blacks is three times that for white infants (Greenwood et al., 2020).

**Figure 1:**
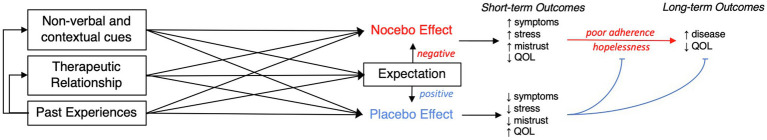
QOL is quality of life, a measure assessed by many treatment satisfaction surveys. Non-adherence, stress, or poor feelings of quality of life could occur unrelated to these factors. Though there are other mechanisms by which non-verbal and contextual cues and past experiences mediate outcomes, one suggested mechanism is that expectation in part mediates these inputs. For example, experience of racism in a clinical encounter could lead to mistrust of that physician and reduce adherence to the treatment suggested by that physician without the influence of expectations. However, the next time that patient returns to a clinician’s office for a medical problem, they might be wary of the potential for a negative encounter, which could then be mediated by expectations, following the paths in the figure.

There are multiple factors that contribute to these inequities, including structural racism and its downstream effects on socioeconomic status and access to healthcare. On an individual level, the clinical encounter is the setting in which many of these streams of influence converge. It is also the setting in which placebo effects can have profound benefits and nocebo effects can seed their greatest harm (Figure 1). There is evidence for discrimination in the clinical encounter that gives rise to unequal care. For example, race of the patient has been shown to significantly impact nurses’ pain-treatment decisions (Hirsh et al., 2010). The implicit bias of clinicians has been suggested to negatively impact their clinical relationships with Black patients, and therefore the care of Black patients in general (Blair et al., 2013). A study of 287 medical residents measured implicit bias and demonstrated that physicians whose scores demonstrated implicit bias against Black patients and implicit adherence to the stereotype that Black patients were less cooperative with medical procedures treated Black and White patients differently (Green et al., 2007). Specifically, these physicians had a higher likelihood of treating White patients with thrombolysis, a treatment for blood clots, as compared to Black patients. In fact, as physicians’ pro-White unconscious biases increased, “so did their likelihood of treating White patients and not treating Black patients with thrombolysis,” indicating unequal treatment related to their biases. Racial and ethnic inequities in care exist in the clinical encounter, giving rise to unequal outcomes and physiological responses to discrimination. Further research is necessary to assess the overlap in neurological correlates of discrimination and nocebo response and to subsequently take steps to improve clinical outcomes.

The absence of factors that promote placebo effects or the presence of factors that induce nocebo effects can have immediate and long-term negative impacts on the patient. Critically, these factors can erode trust and increase perceived discrimination, creating credible expectations that can not only compromise future clinical encounters, but also undermine treatment adherence. It is important to reiterate that these expectations are not deliberate, conscious, or the fault of the patient; rather, these expectations arise as learned conclusions after the direct experience or awareness of others’ experiences of patterns of suboptimal, dismissive, or low-quality care. Substantial evidence shows that patients expect to receive unequal treatment based on their race or ethnicity (Blendon et al., 2007). From evidence that shows anticipation of negative outcomes can influence future outcomes, it follows that expectations of this nature could perpetuate the occurrence of the feared outcome. Unfortunately, far too often “placebogenic” factors are absent and “nocebogenic” factors are present in the clinical encounters of Black Americans compared to those of White Americans.

## The Clinical Encounter

*“A cold*, *uncaring*, *disinterested*, *and emotionless physician will encourage a nocebo response*. *In contrast*, *a caring*, *empathetic*, *physician fosters trust*, *strengthens beneficent patient expectations*, *and elicits a strong placebo response*. *[…] The doctor*, *the nurse*, *the healthcare provider are the most valuable resources for healing patients*.*”* (Olshansky, 2007).

Placebo research has identified the clinical encounter and therapeutic relationship as a key mediator of placebo effects (Figure 1). In turn, the therapeutic benefits of a positive patient-clinician relationship and their downstream effects on improved adherence and clinical outcomes are well established (Schoenthaler et al., 2012). Specifically, studies have isolated physician warmth and empathy as key components of a positive therapeutic encounter and physician coldness and incompetence drivers of negative outcomes.

In order to reduce the influence of nocebo on outcomes, experts encourage physicians to build a good relationship with the patient (Barsky et al., 2002). In physician training, the importance and benefits of a positive and effective therapeutic relationship are emphasized. However, health inequities research finds that the likelihood and frequency of positive or satisfactory encounters with physicians are variable and often vary in part depending on the race and ethnicity of the patient. In a study comparing survey responses of 14 racial and ethnic groups, the non-White groups tended to view the quality of the health care they received more negatively than White patients, and many reported that they felt they would not receive the best care if they were sick (Blendon et al., 2007).

### Communication and Warmth

In the placebo literature, positive and effective clinician patient communication is crucial to ensuring adequate patient satisfaction and adherence to medical advice. Communication is a key component of expectations, which in turn drive placebo and nocebo effects. In a now classic placebo/nocebo study, participants rated their pain during a continuous infusion of remifentanil. The participants’ experience of pain varied with the information they were given about the infusion. Specifically, their experience of pain was reduced when they thought they were receiving the potent analgesic, even if the infusion had not yet started, and enhanced when they were told that the infusion was stopped, even if it was continued. Brain images collected during the study revealed that activation in regions of the brain involved in processing pain or associated with placebo effects was influenced by what the participant was told about the infusion (Bingel et al., 2011). These findings have been replicated in the placebo/nocebo literature (Wager and Atlas, 2015; Geuter et al., 2017). Expectations shaped by information, associative learning, social observation, and subtle cues appear to elicit changes in the brain that are associated with an increase or decrease in the experience of pain.

In psychotherapy trials, patient ratings of empathy and alliance rank as top variables that predict outcomes (Wampold, 2015). Other factors, including supportive care and touch can influence patient satisfaction (Lopez-Sola et al., 2019; Reddan et al., 2020). Enhancing the patient-clinician encounter influences the placebo effect significantly (Fuentes et al., 2014). In one of the most cited placebo studies, Kaptchuk et al. (2008) demonstrated that components of the clinical encounter, from the physical exam and answering questions about one’s condition (“observation”), to receiving sham interventions (“limited”), or sham interventions plus demonstrable warm, caring actions (“augmented”) are additive. In the study, the limited group had better outcomes than the observation group, and the augmented group had significantly better outcomes than the other two groups.

In the nocebo literature, poor or limited physician encounters can increase the likelihood for a nocebo reaction to an inert treatment. One such study used a factorial (2 × 2 × 2) between-subjects study design to assess the impact of expectations and physician interaction style (Howe et al., 2017). In the study, a histamine skin-prick test was used to administer an allergic reaction. Then, an inert cream was provided with instructions to expect either alleviation (the cream would decrease the reaction) or worsening (the cream would increase the reaction). The clinician administering the treatment was instructed to display either high or low warmth and high or low competence. The subjects who received the positive information about the cream had significantly smaller wheals than those that received the negative suggestion. Further, the impact of expectation was mediated by physician warmth and competence. Positive expectations delivered by a warm and competent clinician correlated with the smallest wheal size, and these effects were negated when the physician delivered the inert cream with low-warmth/low-competence.

Another recent study of the effect of medical provider facial expressions on pain analgesia following hypothetical painful procedures found that perceived competence and warmth of the clinician based on facial visual information alone predicted patients’ expectations about post-procedure pain and use of medication (Necka et al., 2021). This study emphasized the effect of patients’ initial impression of physicians on their perceptions of their care, which can subsequently alter treatment outcomes. These findings are consistent with observations that negative information and the absence of warmth and competence can minimize the benefit of a treatment.

Though not a primary focus of the placebo/nocebo literature, validation of patient concerns is an element of the therapeutic relationship that has been shown to influence patient satisfaction with the clinical encounter (Greville-Harris and Dieppe, 2015). One study manipulated the amount of validating language used by a clinician during an encounter to determine the significance of validating language in participant satisfaction. The study demonstrated that participants experiencing an “invalidating” encounter reported lower levels of perceived safety, exhibited higher physiological arousal (measured by heart rate), had increased negative affect, and reported lower willingness to participate in a future study as compared to participants that had encounters with physicians that were validating (Greville-Harris, 2013). In post-visit interviews, invalidated participants reported feeling hopeless and angry, and felt an increased need to avoid particular doctors or treatments entirely. The authors hypothesized that validating language might induce placebo responsiveness, but the results showed that the negative effects of invalidation were more significant and suggest that invalidation could elicit nocebo responses (Greville-Harris and Dieppe, 2015). In a study assessing the impact of physician invalidation on fibromyalgia patients, invalidation correlated with significantly lower quality of life scores on the Quality-of-Life Scale-16 (QOLS-16). Without intervention, constant invalidation of the patient’s experience could have detrimental effects on a patient’s overall sense of wellbeing and psychological and physical health (Lobo et al., 2014).

Racial disparities in communication have been documented and are considered driving factors for poor treatment adherence and negative experiences in the health care setting (Hamel et al., 2021). When recordings of clinic visits were assessed by independent raters, non-White patients were found to experience unfavorable treatment and suboptimal communication compared to their White counterparts. Physicians were found to be 23% more verbally dominant and 33% less engaged in patient-centered communication during medical visits with Black patients as compared to White patients (Johnson et al., 2004). Consistent with these findings, a meta-analysis showed that physicians were less likely to participate in shared decision making or establish rapport with non-White compared to White patients (Ferguson and Candib, 2002).

Black patients are more likely to be invalidated by their physicians. A recent study evaluated language used by physicians in their notes about patients. The study showed that physicians tended to use language implicating lower patient credibility when reporting Black patients’ health concerns in their notes as compared to those of White patients (Beach et al., 2021). According to the 2020 KFF Report, 41% of Black women with a child under the age of 18 stated that there was a time in the last 3 years when a healthcare provider talked down to them or did not treat them with respect (Hamel et al., 2020). Further research has shown that Black patients receive less information than White patients, ask fewer questions, and are less likely to participate in decision making when experiencing lower-quality communication (Shen et al., 2018). Healthcare professionals have also been shown to hold false beliefs about biological differences between White and Black patients that correlate with their treatment decisions (Hoffman et al., 2016; Atlas, 2021). A study assessing whether patient factors affected physicians’ underestimation of patient pain found that physicians are twice as likely to underestimate pain in Black patients (Staton et al., 2007). Black patients reporting moderate to high levels of pain are less likely to receive opioids compared to White patients (Burgess et al., 2014). Further, Black children with appendicitis are less likely to receive opioids for pain relief (Goyal et al., 2015).

The Implicit Association Test (IAT) is a test that quantitatively assesses an individual’s implicit attitudes and beliefs (implicit associations) that they may be unwilling or unable to report. In a study using the IAT to measure clinician implicit race bias and implicit race and compliance stereotyping, high scores (indicating greater bias) were associated with markers of poor communication and care ratings among Black patients in particular (Cooper et al., 2012). Discrimination and stereotyping could explain suboptimal communication between Black patients and their physicians. A study examining the effect of race and socioeconomic status on physicians’ perceptions of patients showed that the race of the patient was associated with the physician’s assessment of the patient’s intelligence, likelihood of risky behavior, likelihood of adherence to medical advice, and the physician’s feelings of affiliation toward the patient (van Ryn and Burke, 2000).

Training physicians to improve their communication with patients is one strategy to address nocebo/placebo effects and inequities in the clinician-patient encounter. Improvement in communication as a result of training was shown to increase the likelihood of patient adherence (Zolnierek and Dimatteo, 2009). Similarly, patient satisfaction is highly correlated with patients’ feelings of involvement in medical decisions and of being treated with dignity (Beach et al., 2005). Positive communication not only benefits the patient; but also a 2016 study similarly showed that relationship-centered communication skills training improved patient satisfaction scores, improved physician empathy and self-efficiency, and reduced physician burnout (Boissy et al., 2016).

### Trust

Trust in the therapeutic relationship is closely tied to communication and is a key mediator of the clinical relationship. In most placebo studies, the importance and influence of a physician is partially attributed to their status as a trusted community member and provider (Benedetti, 2013). Trust is accepted as an important factor in maintaining continuity of care, adherence to treatment, and patient satisfaction, all of which are short-term measures that tend to predict long-term outcomes (Rolfe et al., 2014). In fact, patient feelings of trust in their clinician are associated with pain ratings during an encounter (Losin et al., 2017). On the one hand, trust in physicians has been shown to strongly influence willingness to seek medical care, participate in research, reveal personal information, and adhere to treatment. On the other hand, medical mistrust has been shown to correlate strongly with underutilization of available health services (Hall et al., 2001; LaVeist et al., 2009). These factors are significant, as treatment adherence significantly correlates with patient outcomes; a meta-analysis of studies measuring adherence and outcomes in the general population demonstrated that there is a 26% outcome difference between high and low adherence (DiMatteo et al., 2002).

Mistrust in physicians is both a symptom and cause of suboptimal clinical encounters. Experiencing discrimination can cause mistrust in medical professionals in future encounters, and latent feelings of mistrust can influence adherence and willingness to seek treatment (Hall et al., 2001; LaVeist et al., 2009). Mistrust can be considered a type of negative expectation: an evidence based anticipation of future encounters after negative experiences (Scharff et al., 2010). If a patient experienced discrimination, ambivalence, or suboptimal care from physicians in past experiences, they might be conditioned to anticipate suboptimal encounters in the future.

There is evidence that people who experience discrimination in daily life are more likely to have feelings of mistrust toward medical professionals (Williams and Mohammed, 2009; Ojikutu and Stone, 2021). Some studies have suggested that Black patients are more likely to report general medical mistrust than White patients (Boulware et al., 2003). A 2020 report by KFF found that only 59% of Black adults surveyed felt they could trust doctors as compared to 78% of White respondents (Halbert et al., 2006; Hamel et al., 2020). Studies have shown that Black patients generally report less trust in their personal physicians, regardless of reported trust in general information sources (Musa et al., 2009). These differences can also be observed over the course of a single clinical visit. A 2006 study examining the quality of patient-clinician communication found that Black and White patients rated pre-visit trust in physicians statistically similarly, but Black patients reported lower post-visit trust and rated physicians lower on communication, support, and partnering as compared to White patients (Gordon et al., 2006). Significant predictors of post-visit trust included physicians’ and patients’ perceptions of physicians’ communication.

The potential of trust and comfort in improving treatment outcomes was validated by a 2018 study that examined the potential benefit of healthcare interventions based in trusted community settings, in this case, a barbershop. In the study, 319 Black male barbershop patrons with hypertension were randomly assigned to an in-shop pharmacist led intervention or an active control group. Among participants assigned to the barbershop-based intervention, 63.6% had a reduction in their blood pressure to normal levels compared to 11.7% of those in the control intervention (Victor et al., 2018). This result affirms how integrating findings in placebo/nocebo and healthcare disparities research can drive innovative approaches for addressing health inequities in clinical care.

### Racial Concordance and Patient Outcomes in the Clinic

Many of the elements discussed in this review have been shown to be mediated by racial concordance, or lack thereof, in the patient-clinician relationship (Schoenthaler et al., 2012). Racially discordant patient-clinician encounters have been shown to include communication difficulties that may contribute to lower quality (Street Jr. et al., 2007). Indeed, patients of color have been found to be more likely to have suboptimal experiences in the health care setting when the care is being provided by a provider of a different race – what is referred to as a racially discordant patient provider interaction. The important role of racial concordance was illuminated by a review of the literature in 2018, which examined 40 published studies with 66 different analyses of patient-physician communication with Black patients compared to others (Shen et al., 2018). This review found that in the majority of these studies Black patients experienced lower communication quality measured in multiple ways, including less information giving by physicians, less partnership building by physicians, shorter visits, lower total word count, and less eye contact. Importantly, however, racial discordance almost always predicted poorer communication and communication was better with racially concordant patient-physician interactions.

A number of recent studies have confirmed the positive effect of racially concordant patient-physician encounters and relationships (Cooper-Patrick et al., 1999; Laveist and Nuru-Jeter, 2002; LaVeist et al., 2003; Meghani et al., 2009; Alsan et al., 2019; Ma et al., 2019; Takeshita et al., 2020). They found that patients of color have greater trust, are more likely to receive appropriate treatment and accept indicated screenings, and thus have better outcomes, when they see physicians of their own race. Simulation studies support these findings (Losin et al., 2017). Some studies have found that racial concordance improves pain scores in studies of acute pain (Anderson et al., 2020). Recent studies have investigated the effect of the race of the speaker in media distribution of COVID-19 information (Torres et al., 2021), and a recent study by Alsan et al. (2021) found that Black and Latinx patients are more likely to trust COVID-19 information when it comes from a physician of their own culture. While it is interesting and important that racially concordant patient-provider relationships mitigate the potential harm that patients of color suffer in the health care system, it is not a solution that is scalable. Given that Black and Latinx physicians together comprise approximately 11% of U.S. physicians (Colleges, 2018), it is a given that most Black and Latinx patients will continue to experience racially discordant patient provider relationships. These results point to the importance of pursuing further empirical research to identify which component of the race/ethnic concordant clinical encounter mediates positive outcomes to improve all patient encounters.

## Neurobiological and Physiological Correlates of Placebo/Nocebo and Discrimination

### Neurobiological Correlates of Placebo/Nocebo

In the past 2 decades, neuroimaging studies have begun to elucidate the neurobiological basis and underlying mechanisms of placebo and nocebo effects. In laboratory-based studies, subjects are exposed to an aversive stimulus (e.g., heat pain or electric shock) and then given an inert treatment (e.g., a saline infusion or inert cream) with the instruction to expect alleviation (i.e., “this will reduce your pain”) or worsening of the pain (i.e., “this will increase your pain”). Association between treatment and its effect can also be induced by conditioning through repeated pairing of a painful stimulus and a treatment to guide the experience of pain (Atlas, 2021).

Neuroimaging during these laboratory procedures implicates signaling of opioids and dopamine. Other neurotransmitters and hormones are likely involved, including vasopressin, which influences social behavior and cortisol levels in humans (Colloca et al., 2016). Though nocebo and placebo appear phenotypically opposite, nocebo is hypothesized to recruit another neurotransmitter signaling cascade, the cholecystokinin (CCK) pathway (Tracey, 2010). The CCK signaling pathway has been associated with anxiety disorders and acute episodes of anxiety or stress. Pain related studies of nocebo have found that nocebo is associated with anticipatory anxiety (Atlas, 2021) and hypothalamic-pituitary-adrenal (HPA) axis activity (Johansen et al., 2003), as measured by levels of cortisol in the blood.

In a 2006 neurobiological study of nocebo, participants were exposed to acute arm pain and given either diazepam (a benzodiazepine), proglumide (a CCK-antagonist), or no treatment (Benedetti et al., 2006). To measure changes in HPA-axis activity, blood cortisol levels were obtained. Diazepam, a medication often used to treat anxiety, blocked both nocebo hyperalgesia and HPA-hyperactivity. Proglumide blocked nocebo hyperalgesia but did not block HPA-axis hyperactivity. Importantly, neither drug showed analgesic influence on the pain itself, but rather affected the nocebo related increase in pain ratings. These results suggested differential involvement of the two systems, but point to the involvement of both stress and anxiety HPA-axis related mechanisms in the brain in response to nocebo. Although neuroimaging has identified these and other mechanisms in placebo/nocebo research, they are not specific enough to show reliable correlations to racial healthcare inequities. More neurobiological research is needed to address the significance of the overlap between nocebo pathways and the physiological correlates of the experience of racial and ethnic discrimination in clinical encounters.

### Neurobiological and Physiological Correlates of Discrimination

Experiencing discrimination in the clinic or in the broader societal context can influence levels of trust and expectations of treatment in patients. In the literature, exposure to discrimination or self-reported experience of discriminatory encounters is often referenced as “perceived discrimination,” and has been shown to mediate the number of patients’ experiences of discrimination and feelings of medical mistrust (Hammond, 2010). Perceived discrimination is associated with a variety of negative health consequences, including a higher risk of mental health conditions and a decrease in well-being, self-perceived health, and mortality (Barnes et al., 2008; Williams and Mohammed, 2009; Todorova et al., 2010; Straiton et al., 2019). Heat related pain has been shown to be associated with perceived racial discrimination in Black, but not White, study participants (Goodin et al., 2013). A neuroimaging study conducted in 2020 found that pain ratings in response to a thermal pain stimulus were mediated by perceived discrimination and by brain activity in regions previously associated with discrimination, pain ratings, and trust in the experimenter (Losin et al., 2020). This association was observed in self-identified African American participants, but not among Hispanic or non-Hispanic White participants.

Exposure to discrimination has also been shown to cause downstream stress related neurobiological effects that might influence long-term downstream health outcomes (Pascoe and Smart Richman, 2009). Some of these physiological effects overlap with neurobiological nocebo mechanisms. Activation of the HPA-axis has been shown to be a correlate of stress and experienced discrimination. While activation of the HPA-axis and release of cortisol are an important adaptation to allow humans to respond to stressful or dangerous situations, sustained high concentrations of cortisol can cause long term damage and dysregulation of the HPA-axis, and has even been associated with increased rates of cardiovascular disease (Lockwood et al., 2018). Black patients have higher rates of fatal cardiovascular disease than White patients; in 2018, Black Americans were 30% more likely to die from heart disease than White Americans (OMH, n.d.).

## Discussion

### Ways to Improve Empirical Research to Assess the Impact of Nocebo and Placebo Effects in Clinical Care

The literature suggests that there are important links between nocebo, placebo, and health inequities in the clinical encounter, though there is a dearth of empirical studies assessing this relationship (Friesen and Blease, 2018). Research studying the effect of perceived similarity between patients and clinicians shows that this increases the placebo effect, yet the role of race and ethnicity has yet to be studied with the same focus on identifying a biological mechanism. Understanding the biological mechanism that connects suboptimal clinical encounters with poor long-term outcomes and its downstream effects is essential to promoting health equity. Gaining a specific understanding of the components of the clinical encounter that mediate suboptimal outcomes through biological mechanisms is the first step in ensuring equitable care in the encounter itself. To achieve this, multidisciplinary research is called for, in which researchers work together to gain a more holistic understanding of the downstream impacts of racial/ethnic discordance and outcomes in the clinical encounter, and include measures to better capture some of these components (Atlas, 2021).

There already exists an extensive literature examining the relationship between race-discordance in the patient-clinician relationship, patient satisfaction ratings, and health outcomes, but many of these studies lack a biological outcome measure, like neuroimaging or blood cortisol levels. Assessment of these physiological measures could assist in elucidating the relationship between race/ethnicity and placebo and nocebo in the clinical encounter. Neuroimaging, genomic, or physiological studies examining the impact of supportive care, touch, or good communication should also add in race/ethnicity of the patient and clinician as a measure to capture how different groups of patient are affected by some of these components. To this end, studies utilizing artificial “groups” of patients based on survey responses could provide useful insights when it comes to racial concordance in the clinical encounter, but to make these studies more directly applicable to the clinic, it may be useful to survey patients in actual clinical encounters for their feelings of group-connectedness to clinicians.

Nocebo effects are present in placebo control arms of randomized clinical trials, and the potential for information provided during the informed consent to give rise to side effects has been extensively studied in this context. There is potential for race/ethnicity of patients and clinicians to influence the rates of side effects in clinical trials (Burroughs et al., 2002). However, we note two important points that minimize the potential to derive clear associations between race and adverse events in clinical trials. One is that people of color generally are underrepresented in clinical trials, so statistical comparison of these groups might be underpowered. Second, genetic variation could function as a confounder to some differences in this setting (McDowell et al., 2006). Increasing race and ethnicity reporting in studies with large quantities of nocebo responses in the placebo arm of clinical trials could be useful in identifying specific factors that give rise to side effects.

There are some clear examples of studies that investigate both elements of the clinical encounter and physiological changes associated with health inequities. In February 2021, Letzen et al. (2021) investigated differences in placebo hypoalgesia associated with verbal suggestions, and found that non-Hispanic Black individuals tended to report greater pain increases after verbal suggestion of increased pain plus a saline infusion compared to non-Hispanic White individuals. Some researchers already examine neural mechanisms activated during clinical encounters; for example, Jensen et al. (2014) used functional MRI to examine neural activity of the physician during an encounter with a patient. Adding in race or ethnicity of the patient or of the physician as a focus of an experiment of this type could provide useful insight into outcomes of the clinical encounter. Integration of these methods into studies of health inequities, and integration of race/ethnicity as a factor in studies of placebo and nocebo in the clinical encounter, would begin to fill the gap of empirical research assessing the effect of race in mediating outcomes arising from the clinical encounter.

### Practical Ways to Enhance Placebo and Reduce Nocebo Effects in Clinical Care

Mitigating factors that reduce placebo or enhance nocebo effects, particularly in racially/ethnically discordant dyads, can be achieved by modifying behaviors in communication and addressing implicit biases. Overall, emphasis on ensuring a patient-centered clinical encounter is important in improving patient satisfaction and other outcomes. In a study examining racial disparities in communication, the results found that communication quality was improved when physicians’ behavior was matched to that of the patients. For example, physicians could focus on matching patient behaviors of smiling, gazing, and laughter as these behaviors have been shown to elicit a favorable response from patients (Hamel et al., 2021). Studies have also shown that the implicit racial bias of physicians can be decreased significantly by completing the Black-White IAT during the first and last semester of medical school (van Ryn et al., 2015). Therefore, implementing unconscious bias training early on in medical education may also prove to be an effective way to prevent discrimination, invalidation, and microaggression from driving racial inequities in patient communication and ultimately health outcomes.

In order to maximize placebo effects and minimize the detrimental effects of nocebo, experts have encouraged clinicians to become familiar with placebo and nocebo effects and to educate their patients about the potential mechanisms of such effects. Similarly, experts advise clinicians to encourage conversation with patients about their needs and expectations about their treatment, and to frame information in a reasonably positive context and avoid negative contextual experiences (Barsky et al., 2002; Colloca and Barsky, 2020).

## Conclusion

In this paper, we examined how healthcare inequities may be driven in part by the absence of placebo and presence of nocebo effects. We focused on components of the patient-clinician relationship that can mediate nocebo and placebo effects disparately across populations. Poor communication, medical mistrust, and perceived discrimination are three components of care that could interact with placebo/nocebo mechanisms to influence downstream effects (Figure 1). Clinicians are not the only sources of negative anticipation; inattentive or discourteous non-medical staff, for example, can contribute to patient feelings of discrimination and lower ratings of patient satisfaction (Tajeu et al., 2015). The short-and long-term effects of health inequities related to clinical care lead to devastating effects that have long term impacts on quality of life. Ideally, the systemic factors seeding the absence of placebo and presence of nocebo effects would be reduced. In the short-term, understanding the underlying biological drivers of these effects is critical to identifying ways to shift and improve or prevent these harmful influences on patients.

The Latin root for “nocebo” is also found in the Hippocratic oath: *primum non nocere*, or “do no harm.” Through suboptimal clinical encounters marked by poor communication and inattention to interpersonal cues that could impact patient feelings of trust, evidence from the existing literature suggests that the potential for nocebo effects could be increased among Black patients. In order to adequately serve patients and minimize the damaging effects of racism, steps should be taken to minimize nocebo and maximize placebo effects. Educating clinicians about their role in mitigating these factors would go a long way in increasing placebo and reducing nocebo in the clinical encounter to ensure the best care for patients. Further, placebo researchers and public health experts must collaborate to address the potential mechanism by which inequity in the clinical encounter affects long-term outcomes, experiences, and perceptions of care.

## Author Contributions

VES, KTH, and HEY contributed to conception and design of the review. HEY wrote the first draft of the manuscript. KTH, VES, SRA, and NC wrote sections of the manuscript. All authors contributed to the article and approved the submitted version.

## Funding

KTH is funded by NHLBI K01HL130625. KTH and HEY are funded by a pilot grant from the Osher Center for Integrative Medicine at Brigham and Women’s Hospital and Harvard Medical School.

## Conflict of Interest

The authors declare that the research was conducted in the absence of any commercial or financial relationships that could be construed as a potential conflict of interest.

## Publisher’s Note

All claims expressed in this article are solely those of the authors and do not necessarily represent those of their affiliated organizations, or those of the publisher, the editors and the reviewers. Any product that may be evaluated in this article, or claim that may be made by its manufacturer, is not guaranteed or endorsed by the publisher.
